# Predictors of implant failure: A comprehensive analysis of risk factors in oral implant restoration for patients with partial defects of dentition

**DOI:** 10.17305/bb.2024.11668

**Published:** 2025-01-15

**Authors:** Dake Linghu, Danna Zhang, Min Liu

**Affiliations:** 1 Oral Implantology Department, Linyi Guoqiang Stomatological Hospital, Yuncheng, China; 2 Department of Orthognathic Surgery and Maxillofacial Trauma, The Third Affiliated Hospital of Air Force Medical University, Xi’an, China

**Keywords:** Dentition defect, oral implant restoration, implant failure events, influencing factors

## Abstract

Implant failure remains a significant challenge in oral implantology, necessitating a deeper understanding of its risk factors to improve treatment outcomes. This study aimed to enhance the clinical outcomes of oral implant restoration by investigating the factors contributing to implant failure in patients with partial dentition defects within two years of treatment. Additionally, the study sought to develop an early risk prediction model for implant failure. A retrospective analysis was conducted on 300 patients with partial dentition defects, dividing them into two groups: a failed implant group and a successful implant group, based on the occurrence of implant failure within two years. General clinical data and condition-specific clinical information were compared between the groups. Multivariate binary logistic regression analysis was used to identify influencing factors, while the predictive effectiveness of the model was assessed using a receiver operating characteristic (ROC) curve. The analysis revealed that factors, such as gender, post-implant smoking, oral hygiene status at the second-year follow-up, tooth position, number of implants, timing of loading, width of keratinized mucosa, and bone quantity significantly influenced the likelihood of implant failure (*P* < 0.05). Among these, post-implant smoking and tooth position were identified as independent risk factors. The area under the curve (AUC) for tooth position was 0.695, indicating low predictive performance. Although tooth position was determined to be an independent risk factor for implant failure within two years, its predictive performance was limited.

## Introduction

According to the Fourth National Oral Disease Epidemiological Survey, 81.7% of individuals aged 65–74 suffer from partial dentition defects or tooth loss [1]. This percentage is expected to rise as the global population ages [2]. Dentition defects are prevalent among middle-aged and elderly individuals, impacting not only oral health but also facial aesthetics. Symptoms include masticatory muscle pain, discomfort, food impaction, and reduced chewing efficiency [3]. These issues often lead to dental jaw deformities, structural changes, and damage to dental tissues [3]. A decline in chewing function can further exacerbate periodontal problems in neighboring teeth, resulting in occlusal dysfunction that adversely affects both oral and mental health. Therefore, personalized treatment plans tailored to specific symptoms are essential [3]. Traditional methods to restore dentition defects include fixed dentures, overdentures, and removable partial dentures, all designed to enhance both aesthetics and masticatory function [4–6]. However, these approaches often have limitations, such as adverse effects on residual teeth and periodontal tissues, reduced stability, and less-than-optimal therapeutic outcomes for some patients [4–6]. Advances in oral treatment technology have introduced implant-based solutions, which involve placing implants in the alveolar bone at the affected site. Once the upper-end restoration is attached, this approach offers a minimally invasive procedure with stable retention and improved aesthetics. Implant-based restorations minimize damage to adjacent teeth, provide effective fixation, and are increasingly favored by patients due to their advantages over traditional methods [7].

Research has indicated that unfavorable outcomes following implant restoration surgery can be attributed to various factors, such as suboptimal surgical techniques, poor bone quality, unsuitable implant sites, infections, inadequate prosthesis design, and occlusal trauma [8]. Numerous studies have focused on identifying risk factors that influence the longevity of dental implants [9–12]. While existing research has highlighted factors contributing to implant restoration failures in cases of partial dentition defects, the findings remain inconsistent, and a universally accepted early risk prediction model has yet to be developed. Furthermore, the determinants of implant restoration failure seem to differ significantly across countries and regions, underscoring the necessity for more comprehensive and localized investigations. In this study, we aimed to evaluate the prevalence and determinants of implant failure events in patients with partial dentition defects within a two-year period following oral implant restoration. Additionally, we sought to develop an early risk prediction model to improve the prognosis and management of implant-related complications.

## Materials and methods

### Patients

This study retrospectively analyzed 300 patients with partial dentition defects who were treated at our hospital between January 1, 2019, and December 31, 2021. The follow-up period lasted two years, during which patients attended annual visits. We reviewed prior implant records and imaging systems to collect general clinical data and relevant information. Based on implant outcomes within the two-year follow-up, patients were categorized into two groups: the failed implant group and the successful implant group. Inclusion criteria: Patients met the diagnostic criteria for dental arch defects as defined in the Clinical Disease Diagnosis and Efficacy Judgment Standards [13]; they underwent successful implant placement and subsequent restoration procedures in our hospital’s specialized Dental Implant Department, with accessible clinical records and radiographic materials. Additionally, patients were aged ≥18 years, demonstrated good compliance with follow-up visits, and had two implants placed in adjacent tooth positions within the same anatomical area. For these cases, restoration was performed using a bridging crown. If two implants were placed in other circumstances, a single crown was used for restoration. Patients were required to have no significant periodontal inflammation. Exclusion criteria: Patients were excluded if they had single-tooth implant-supported bridges or if the number of implants placed did not match the number of restorations. Other exclusions included the use of bone grafting materials, autogenous bone, or biological membranes during surgery; cases where assisted bone compression techniques or maxillary sinus floor elevation procedures were performed (e.g., when insufficient bone volume required the use of synthetic bone substitutes, demineralized bone grafts, or collagen membranes to enhance osseointegration). Exclusion criteria also applied to patients whose implant surgery or crown restoration was performed by a junior doctor, those with complications from implanted devices that were resolved without device removal, patients with uncontrolled diabetes or systemic diseases, individuals who received head and neck radiotherapy or bisphosphonates during follow-up, patients with ≥ three intraoral implants, and individuals with psychiatric or psychological conditions affecting treatment adherence.

### Indicator collection

Patients were followed up annually after implant restoration surgery, with the occurrence of implant failure events recorded during the second-year follow-up. Implant failure events were categorized as follows [14]: Infection: Presence of purulent discharge, no mobility or slight mobility of the implant, and no significant bone loss observed on radiographs. Peri-implantitis: Recurrent purulent discharge, no mobility or slight mobility of the implant, and bone loss of 2–4 mm on radiographs. Lack of osseointegration: Absence of purulent discharge and significant radiographic abnormalities, but the implant exhibits significant mobility. Fracture or separation: Fracture or detachment of the implant and abutment. Other causes: Including patient-reported pain or psychological inability to accept the implant. For cases where the implant has surpassed the clinical osseointegration period but exhibits significant mobility without purulent discharge or radiographic abnormalities, the failure is attributed to a lack of osseointegration. Conversely, if purulent discharge and bone loss are present, the condition is classified as peri-implantitis, while purulent discharge without significant bone loss is categorized as an infection. Patients were also assessed annually for general clinical data during the second-year follow-up. The general clinical data collected included: gender, age at surgery, surgeon qualifications, presence of diabetes, cardiovascular disease, or osteoporosis, oral hygiene habits (e.g., brushing and mouth rinsing), history of gingival bleeding, smoking and drinking habits after implant placement, and overall oral hygiene status at the second-year follow-up. Additionally, clinical condition-related information was gathered during the second-year follow-up. This information included: jaw position, tooth position, implant type, number, length, and diameter of implants, timing of implant placement and loading, timing of antibiotic use, width of keratinized mucosa, fixation method, occlusal contact, plaque index, calculus index, and bone quantity. Note on bone quantity: Bone quantity refers to the total amount of bone substance within the skeletal framework, an important metric for evaluating bone density and strength. For this study, QCT was used to measure bone density in participants, and these measurements were converted into quantifiable bone quantity values.

### Ethical statement

The study protocol was approved by the Ethics Committee of our hospital (2021-K09). All participants or their relatives signed the written informed consent before recruitment.

### Statistical analysis

Statistical analysis was performed using IBM SPSS Statistics Version 27.0.1 (SPSS Inc., Beijing, IL, China). Categorical variables were presented as *n* (%). If the data met the criteria of theoretical frequencies greater than five and a total sample size of at least forty, comparisons between groups were conducted using Pearson’s chi-square test. Normality tests for continuous data were first performed using histograms and the one-sample Shapiro–Wilk test. Continuous variables following a normal distribution were expressed as mean ± standard deviation (x- ± s). For inter-group comparisons, if the assumption of homogeneity of variance was met, two independent sample *t*-tests were used. If this assumption was not met, Welch’s *t*-test was applied. Quantitative data with a skewed distribution were expressed as the median (interquartile range), and comparisons between two groups were performed using the Mann–Whitney *U* test for independent samples. The study categorized patients with partial dentition defects into two groups: the failed implant group (coded as 1) and the successful implant group (coded as 0). Statistically significant factors identified through univariate analysis were used as predictor variables to construct a multivariate logistic regression model for early prediction of implant failure events. The model identified significant factors (*P* < 0.05) as independent predictors of implant failure, enabling more accurate risk assessments. Multivariate logistic regression analysis was employed to identify related risk factors and to develop an early risk prediction model for implant failure. A receiver operating characteristic (ROC) curve was used to determine the area under the curve (AUC), Youden index, optimal cutoff value, sensitivity, and specificity of the prediction model and each risk factor, further evaluating their predictive efficacy. According to Yu et al. [15], when the AUC of an ROC curve is between 0.5 and 0.7, it indicates a low predictive effect; between 0.7 and 0.9, a moderate predictive effect; and above 0.9, an excellent predictive effect.

## Results

### The occurrence of implant failure events

Among 300 patients with partial dentition defects who underwent oral implant restoration, 17 experienced implant failure within two years, representing 5.67% of the total. Of these cases, six patients (35.29%) developed peri-implantitis, five (29.41%) experienced infections, two (11.76%) exhibited a lack of osseointegration, three (17.65%) had implant fractures, and one (5.88%) reported pain accompanied by psychological issues related to the implants (Table 1).

**Table 1:** The occurrence of implant failure events

**Variables**	***n* (%)**
Successful implant events	283 (94.33)
Implant failure events	17 (5.67)
Peri-implantitis	6 (35.29)
Infection	5 (29.41)
Lack of osseointegratio	2 (11.76)
Implant fracture	3 (17.65)
Other	1 (5.58)

Results expressed as the number of patients and percentage (%).

### Comparison of general clinical data between the two groups of patients

Among 300 patients with partial defects of dentition, the failure group consisted of 17 cases (5.67%), and the success group consisted of 283 cases (94.33%). Gender (*P* ═ 0.008), smoking after implant placement (*P* ═ 0.049), and oral hygiene status at the second-year follow-up of oral implant restoration (*P* ═ 0.026) were factors affecting the occurrence of implant failure events within two years of oral implant restoration in patients with dentition defect. Brushing habits, surgical age, surgeon qualifications, diabetes, cardiovascular disease, osteoporosis, mouth rinsing habits, history of gingival bleeding, and drinking after implant placement were not factors affecting the occurrence of implant failure events within two years of oral implant restoration in patients with dentition defect (*P* > 0.05; Table 2).

**Table 2:** Comparison of general clinical data between the two groups of patients

**Variables**	**Failed implant group (*n* ═ 17)**	**Successful implant group (*n* ═ 283)**	**χ^2^/t value**	***P* value**
Gender (Male/Female)	9/8	113/170	6.957	0.008
Surgical age (Year)	43.56 ± 9.26	42.78 ± 8.94	0.732	0.465
Surgical physician qualifications (High-seniority expert/Low-seniority expert)	105 (61.05)/67 (38.95)	75 (58.59)/53 (41.41)	0.184	0.668
Diabetes (Yes/No)	20 (11.63)/152 (88.37)	12 (9.38)/116 (90.62)	0.391	0.532
Cardiovascular disease (Yes/No)	51 (29.65)/121 (70.35)	34 (26.56)/94 (73.44)	0.345	0.557
Osteoporosis (Yes/No)	9 (5.23)/163 (94.77)	5 (3.91)/123 (96.09)	0.290	0.590
Brushing habits (1 times/d/≥ 2 times/d)	38 (22.09)/134 (77.91)	24 (18.75)/104 (81.25)	0.500	0.479
Mouth rinsing habits (Often/Almost not)	94 (54.65)/78 (45.35)	76 (59.38)/52 (40.62)	0.667	0.414
History of gingival bleeding (Yes/No)	79 (45.93)/93 (54.07)	50 (39.06)/78 (60.94)	1.412	0.235
Smoking after implant placement (Yes/No)	4/13	20/263	3.880	0.049
Drinking after implant placement (Yes/No)	134 (77.91)/38 (22.09)	89 (69.53)/39 (30.47)	2.698	0.100
Oral hygiene status at the second-year follow-up of oral implant restoration (Excellent/Poor)	12/5	226/57	4.610	0.032

Results expressed as means ± SD, or the number of patients and percentage (%); surgical physician qualifications (high-seniority expert: senior professional title ≥5 years; low-seniority expert: senior professional title <5 years); oral hygiene status at the second-year follow-up of oral implant restoration (excellent: teeth were clean, gums had normal color, no bleeding when brushing; poor: soft plaque on teeth, tartar, abnormal color of gums, bleeding when brushing).

### Comparison of clinical information related to the condition between the two groups of patients

The factors influencing the occurrence of implant failure events within two years of oral implant restoration in patients with dentition defects include tooth position (*P* < 0.001), number of implants (*P* ═ 0.035), timing of loading (*P* ═ 0.001), width of keratinized mucosa (*P* ═ 0.028), and bone quantity (*P* ═ 0.036). The jaw position, implant type, length of implants, diameter of implants, timing of implant placement, timing of antibiotic use, fixation method, occlusal contact situation, plaque index, and calculus index were not factors affecting the occurrence of implant failure events within two years of oral implant restoration in patients with dentition defect (*P* > 0.05; Table 3).

**Table 3:** Comparison of clinical information related to the condition between the two groups of patients

**Variables**	**Failed implant group (*n* ═ 17)**	**Successful implant group (*n* ═ 283)**	**χ^2^/t value**	***P* value**
Jaw position (Maxillary position/Mandibular position/Maxillary position + Mandibular position)	79 (45.93)/91 (52.91)/2 (1.16)	60 (46.88)/67 (52.34)/1 (0.08)	0.327	0.954
Tooth position (Anterior dental regions/Posterior dental regions)	8/9	23/260	22.200	<0.001
Implant type (Bone level implant/Soft tissue level implant)	144 (83.72)/28 (16.28)	110 (85.94)/18 (14.06)	0.278	0.598
Number of implants (1/2)	15/2	272/11	4.460	0.035
Length of implants (< 10 mm/≥ 10 mm)	26 (15.12)/146 (84.88)	16 (12.50)/112 (87.50)	0.417	0.518
Diameter of implants (< 3.5 mm/≥ 3.5 mm)	28 (16.28)/144 (83.72)	15 (11.72)/113 (88.28)	1.243	0.265
Timing of implant placement (Immediately/Early/Late)	9 (5.23)/68 (39.53)/95 (55.23)	10 (7.81)/63 (49.22)/55 (42.97)	4.555	0.103
Timing of loading (Early/Late)	13/4	257/26	11.181	0.001
Timing of antibiotic use (Preoperative/ Postoperative)	115 (66.86)/57 (33.14)	90 (70.31)/38 (29.69)	0.404	0.525
Width of keratinized mucosa (≥ 1 mm/< 1 mm)	11/6	223/60	4.820	0.028
Fixation method (Bonding retention/Screw retention)	154 (89.53)/18 (10.47)	117 (91.41)/11 (8.59)	0.294	0.588
Occlusal contact situation (Good/Poor)	103 (59.88)/69 (40.12)	88 (68.75)/40 (31.25)	2.494	0.114
Plaque index (None/< 1/3/≥ 1/3 & ≤ 2/3/>2/3)	12 (6.98)/115 (66.86)/43 (25.00)/2 (1.16)	10 (7.81)/99 (77.34)/18 (14.06)/1 (0.08)	5.812	0.110
Calculus index (None/< 1/3/≥ 1/3 & ≤ 2/3/>2/3)	11 (6.40)/91 (52.91)/69 (40.12)/1 (0.06)	10 (7.81)/67(52.34)/47 (36.72)/4 (3.13)	3.135	0.376
Bone quantity (Adequate/Insufficient)	10/7	207/76	4.408	0.036

Results expressed as means ± SD, or the number of patients and percentage (%); timing of implant placement (immediately: implantation on the day of tooth extraction; early: 4–16 weeks; late: >16 weeks); timing of loading (early: 1–8 weeks post-implantation; late: >8 weeks).

### Variable assignment

We grouped the patients based on whether they experienced an implant failure within two years (assigning the failed implant group a value of 1 and the successful implant group a value of 0). Statistically significant factors identified in the single-factor analysis were then assigned as independent variables (Table 4).

**Table 4:** Variable assignment

**Related variables**	**Assignment**
Gender (Male/Female)	Female ═ 0, Male ═ 1
Smoking after implant placement (Yes/No)	No ═ 0, Yes ═ 1
Oral hygiene status at the second-year follow-up of oral implant restoration (Excellent/Poor)	Poor ═ 0, Excellent ═ 1
Tooth position (Anterior dental regions/Posterior dental regions)	Posterior dental regions ═ 0, Anterior dental regions ═ 1
Number of implants (1/2)	2 ═ 0, 1 ═ 1
Timing of loading (Early/Late)	Late ═ 0, Early ═ 1
Width of keratinized mucosa (≥ 1 mm/< 1 mm)	<1 mm ═ 0, ≥1 mm ═ 1
Bone quantity (Adequate/Insufficient)	Insufficient ═ 0, Adequate ═ 1

### Analysis of implant failure factors within two years in partially dentate patients

Multivariate binary logistic regression analysis revealed that smoking after implant placement (*P* ═ 0.044) and tooth position (*P* ═ 0.007) were independent risk factors for implant failure within two years following oral implant restoration in patients with partial dentition defects (Table 5).

**Table 5:** Multivariate logistic regression analysis for identifying risk factors of implant failure events occurred within two years of oral implant restoration in patients with dentition defect

**Factors**	**Partial regression coefficient (β)**	**Standard error**	***Wald*** **χ^2^ value**	***P* value**	**OR value**	**95% CI value**	
						**Lower limit**	**Upper limit**
Gender (Male/Female)	0.043	1.422	0.001	0.976	1.044	0.064	16.951
Smoking after implant placement (Yes/No)	−1.897	0.940	4.074	0.044	0.150	0.024	0.946
Oral hygiene status at the second-year follow-up of oral implant restoration (Excellent/Poor)	2.335	1.538	2.306	0.129	10.333	0.507	210.586
Tooth position (Anterior dental regions/Posterior dental regions)	4.787	1.263	14.375	<0.00 1	120.000	10.101	1425.623
Number of implants (1/2)	−0.310	1.076	0.083	0.773	0.733	0.089	6.041
Timing of loading (Early/Late)	−1.419	1.264	1.259	0.262	0.242	0.020	2.884
Width of keratinized mucosa (≥1 mm/<1 mm)	−1.674	1.548	1.170	0.279	0.188	0.009	3.895
Bone quantity (Adequate/Insufficient)	−1.771	1.440	1.512	0.219	0.170	0.010	2.861

### The predictive value of the independent impact factors for implant failure events occurred within two years of oral implant restoration in patients with partial defects of dentition

**Figure 1.**
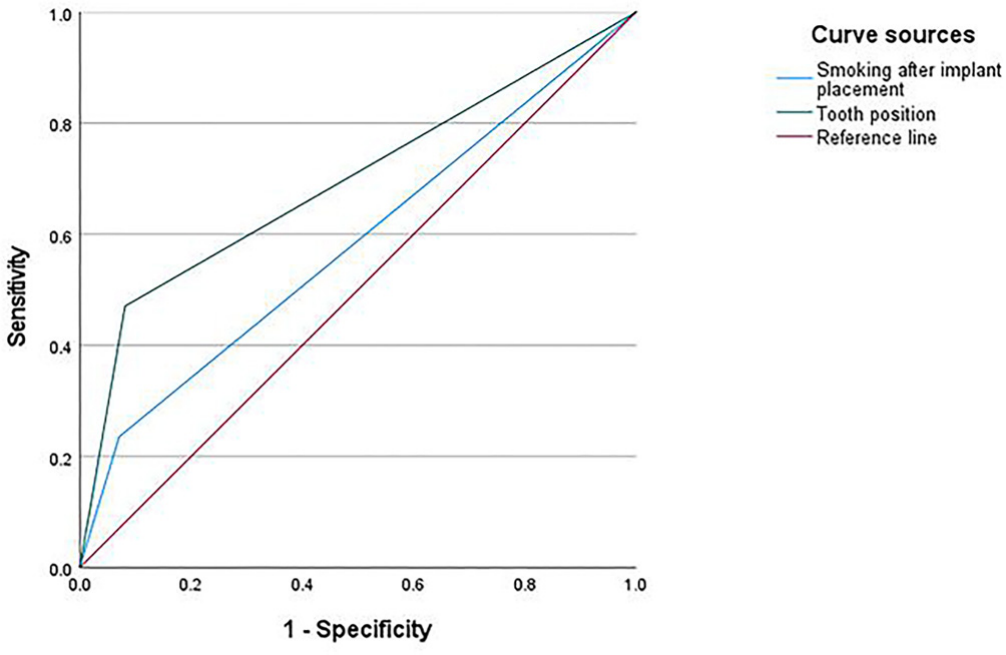
**ROC curve of the performance of the independent impact factors for implant failure events occurred within two years of oral implant restoration in patients with dentition defect**. ROC: Receiver operating characteristic.

ROC curves were plotted for the independent impact factors to assess their predictive performance. The results showed that the AUC for smoking after implant placement was 0.582, which is greater than 0.5 but indicates only poor predictive power. In contrast, the AUC for tooth position was 0.695, suggesting low predictive power (Table 6, Figure 1).

**Table 6:** The predictive value of the independent impact factors for implant failure events occurred within two years of oral implant restoration in patients with dentition defect

**Predictive variables**	**AUC**	**95% CI**	***P* value**	**Youden index**	**Cutoff value**	**Sensitivity**	**Specificity**
Smoking after implant placement (Yes/No)	0.582	0.429∼0.736	0.254	0.164	0.500	0.235	0.929
Tooth position (Anterior dental regions/Posterior dental regions)	0.695	0.542∼0.847	0.007	0.390	0.500	0.471	0.919

AUC: Area under the curve.

## Discussion

This study examined 300 patients with partial dentition defects who underwent oral implant restoration. Within two years, 17 patients experienced implant failure, accounting for 5.67% of the total. Of these, six patients (35.29%) developed peri-implantitis, five (29.41%) experienced infection, two (11.76%) lacked osseointegration, three (17.65%) had implant fractures, and one (5.88%) encountered another failure type involving pain and psychological distress, leading to voluntary implant removal. Yang et al. [14] analyzed 89 patients with implant failures, identifying 38 cases of peri-implantitis, 28 infections, nine instances of osseointegration failure, 12 implant fractures, and two other failure types. These findings align with the present study’s outcomes. A 2019 study reported peri-implant disease incidence at 50.72% among patients with fixed restorations for partial dentition defects [10]. Xiaodong et al. [16] observed a 22.61% overall complication rate post-implant restoration in such patients. Wong et al. [17] studied 45 patients with dental implant restorations and found adjacent tooth loss incidence at 65% and food impaction at 40%. Similarly, Shen et al. [18] reported that 19% of patients experienced prosthesis loosening or breakage within six months, while 54% of patients who used prostheses for over six months encountered complications. The above findings, along with the results of this study, suggest a high probability of implant failure in patients with partial dentition defects following oral implant restoration. These results underscore the need for early risk prediction, timely interventions, and tailored restoration plans to reduce implant failure rates. Moreover, optimizing post-implant nursing care is crucial to enhance restoration outcomes and patient prognoses. Therefore, investigating the factors influencing implant failure is essential to guide clinical nursing practices and improve relevant care protocols.

Our research identified gender as a risk factor for implant failure, with male patients being 5.367 times more likely to experience implant failure compared to female patients (*P* < 0.001). This disparity may be attributed to the higher prevalence of certain detrimental habits among males, such as smoking. Smoking introduces nicotine, which reduces oxygen absorption by the gums, increases gum sensitivity to pathogenic bacteria and their toxins, stimulates bone resorption, and ultimately hinders the integration of the implant with bone tissue, thereby raising the risk of implant failure [19, 20]. The study demonstrated that smoking after implant placement was an independent risk factor for implant failure events within two years of oral implant restoration in patients with partial dentition defects. Smoking can suppress immune function, encourage the growth of anaerobic bacteria in the oral cavity [21, 22], accelerate plaque accumulation, and promote bone resorption in the implant socket [23]. Numerous studies have reported that smoking elevates the risk of peri-implantitis, with a significant increase in the prevalence of both periodontitis and peri-implantitis among smokers [24–27]. Some scholars observed that the implant failure rate among smokers could reach as high as 20% [28]. Furthermore, smokers face double the risk of implant failure compared to non-smokers [28, 29]. Torsten et al. [30] found that the implant failure rate correlates with the duration and intensity of smoking. For instance, patients who smoke more than 20 cigarettes daily face a 20% higher risk of implant failure than those who smoke less [31]. In a retrospective cohort study involving 295 patients and 1033 implants conducted at the University of Barcelona Dental School, Rodriguez-Argueta et al. [26] reported that smokers had an elevated risk of complications, including implant loss, mucositis, and peri-implantitis. Similarly, Ann-Marie et al. [32] observed that smokers are more susceptible to peri-implant diseases, including mucositis, significant bone loss (≥3 threads), and peri-implantitis. However, conflicting results exist. Koldsland [33] and Marrone [34] found no significant correlation between smoking and peri-implantitis in their studies. Such discrepancies may stem from variations in smoking intensity and duration among study participants or differences in the implant systems used. Additionally, male patients tend to exhibit poorer oral hygiene awareness and compliance compared to females, as well as limited knowledge about oral health. Retrospective studies have suggested that, over the long term, there is no significant difference in implant success rates between males and females [35]. However, for early implant failure, detrimental habits remain key influencing factors. Postoperative recommendations emphasize that patients should actively cooperate, strive to correct harmful habits, and maintain optimal oral health and hygiene to improve implant outcomes. Research has shown that the timing of implant placement is not a significant risk factor for implant surgery [36, 37]. However, other studies have indicated that implants placed in the late stage achieved the highest success rate (100%), outperforming early placement (96.15%), and immediate placement (90.32%) [38]. These discrepancies in study outcomes are attributed to varying definitions of implant timing across different studies, with overlapping time frames. This study adopted the definitions of implant timing and loading timing provided in the 2021 EAO Consensus Meeting - Group 1 Summary and Consensus Statements [39]. Early loading of implants has demonstrated stable long-term outcomes with a low failure rate, significantly reducing the overall treatment time for implant restoration [12]. Notably, early loading facilitates micro-movements of the implants, which can promote bone integration and, in some cases, even surpass the bone integration effects observed under non-loading conditions [40]. This study further observed that loading timing serves as a protective factor against implant failure within two years of oral implant restoration in patients with partial dentition defects. Early loading was associated with a substantially lower risk of implant failure, with a hazard ratio of 0.255 compared to late loading (*P* ═ 0.021). These findings emphasize the importance of loading implants as early as possible to reduce implant failure rates, enhance restorative outcomes, and improve overall implant success rates. Bone quantity was also identified as a protective factor against implant failure within two years of restoration in patients with partial dentition defects. This could be due to the close relationship between bone quantity, the implantation process, and the bone integration of dental implants. Sufficient bone quantity is widely recognized as a prerequisite for successful implant-supported restorations [16]. Research by Li and colleagues demonstrated that insufficient bone quantity can lead to decreased bone density before surgery. Compared to patients with adequate bone quantity, those with deficiencies are at a higher risk of experiencing poor restoration stability, delayed bone integration, and failure in bone remodeling. These challenges can result in bone resorption, loosened or detached implants, and an increased risk of complications [41]. Thus, assessing bone density in patients with bone deficiencies and implementing appropriate preoperative strategies are critical to ensuring successful implant restoration outcomes [42]. Oral hygiene status at the two-year follow-up was another significant factor influencing implant failure in patients with partial dentition defects. Poor oral hygiene can promote plaque accumulation and biofilm formation, which trigger inflammation around dental implants. If untreated, this inflammation may escalate to implant-related infections, pain, and eventual implant failure [43–45]. Studies by Ferreira [46] and Ann-Marie [32] confirmed a causal link between plaque accumulation and the onset of peri-implant mucositis. Additionally, a ten-year retrospective study by Gianluigi et al. [47] highlighted the correlation between poor oral hygiene and bone loss around dental implants. Research has also shown that implant sites with >30% plaque accumulation and bleeding upon probing are at higher risk for peri-implant mucositis and peri-implantitis [48]. The full-mouth plaque index is positively correlated with the incidence of peri-implant diseases, and severe oral hygiene neglect (mean full-mouth plaque index ≥2) is strongly associated with peri-implantitis (OR ═ 14.3; 95% CI 2.0–4.1) [46]. Microorganisms from the periodontal pockets of remaining natural teeth in partially edentulous patients can also migrate to the peri-implant sites, further contributing to complications [49]. Daniel et al. [50] found that oral hygiene habits, such as brushing frequency and duration significantly impact the incidence of peri-implantitis. For instance, patients who brush ≥2 times a day experience significantly fewer cases of peri-implantitis compared to those who brush only once. Patients who brush for ≥ three minutes have a significantly lower incidence of peri-implantitis than those who brush for < three minutes [51]. The clinical success of dental implants is highly dependent on good oral hygiene; however, maintaining proper hygiene can be challenging for many patients [52]. Therefore, oral surgeons should educate patients on the importance of oral hygiene, design prosthetic restorations that facilitate self-maintenance, and teach proper hygiene techniques to optimize implant success and longevity.

This study concluded that tooth position is an independent factor influencing the occurrence of implant failure events within two years of oral implant restoration in patients with partial dentition defects. The higher failure rate in the anterior region might be attributed to a thinner bone cortex, the inclination of anterior teeth, and the predominantly non-axial transmission of biting forces, which create tilting forces and torques. These forces can compromise the initial stability of the implant, leading to bone resorption and eventual implant loss. Supporting this, Hee-Won et al. [53] analyzed 3755 patients (6385 dental implants) and observed a slightly higher failure rate in the anterior region (2.17%) compared to the posterior region (1.62%), consistent with this study’s findings. Factors, such as lower cancellous bone content, poor blood supply, narrower alveolar crest width, and horizontal bone deficiencies in the anterior region may also contribute to the increased risk of implant failure [14]. Although theoretically, using two implants for a bridge could help distribute occlusal forces, this study found that the risk of implant failure events was significantly higher with two implants compared to a single implant (*P* ═ 0.035). This could be due to one implant generating torque on the other, destabilizing the system and ultimately leading to failure. Previous studies have similarly reported that two implants increase tension and pressure zones around the implant and surrounding bone, adversely affecting implant stability [54]. A meta-analysis showed that two implants compared to one resulted in more severe bone resorption at the implant margin and a higher failure rate [55]. While multiple implants may better handle axial occlusal forces, they appear less effective in managing lateral occlusal forces [56]. These findings align with this study and emphasize the need for careful planning and monitoring of cases requiring multiple implants to minimize failure risks and improve prognosis. The keratinized mucosa surrounding dental implants functions similarly to attached gingiva around teeth, maintaining stability and preventing tissue attachment loss. However, its correlation with peri-implant diseases remains controversial. While some studies, such as those by Hanisch et al. [57] and Strub et al. [58], found no significant effect of keratinized mucosa on peri-implant tissue health, this study identified the width of keratinized mucosa as a factor influencing implant failure events within two years. Supporting this, other studies have shown that insufficient keratinized mucosa can exacerbate peri-implant mucosal inflammation and plaque accumulation, [59–61] leading to bone loss [62, 63] or attachment loss [64]. Insufficient keratinized mucosa increases the risk of mucosal recession, particularly in the aesthetic zone, where adequate keratinized mucosa is recommended [59–61]. Ann-Marie et al. [32] associated the presence of keratinized mucosa with reduced mucositis and bone loss, while Ausra et al. [65] noted its protective role in decreasing peri-implantitis incidence. Retrospective analyses have also linked insufficient keratinized mucosa—particularly in posterior implants—to plaque accumulation and gingival inflammation [66]. Although no conclusive evidence supports routine soft tissue surgery to increase keratinized mucosa, ensuring good plaque control is critical for implant success. In areas where oral hygiene maintenance is challenging, surgical interventions to enhance keratinized mucosa may be beneficial.

This study sheds light on various factors influencing implant failure, such as gender, smoking after implant placement, oral hygiene status at the second-year follow-up, tooth position, number of implants, timing of loading, width of keratinized mucosa, and bone quantity. Notably, smoking after implant placement and tooth position emerged as independent impact factors. However, certain limitations should be considered. For instance, our analysis did not identify jaw position, implant type, implant length, implant diameter, timing of implant placement, timing of antibiotic use, fixation method, occlusal contact situation, plaque index, or calculus index as significantly affecting implant failure within two years of oral implant restoration in patients with dentition defects. It is possible that some patients may have intentionally withheld information, leading to discrepancies between self-reported data and the actual clinical situation. Additionally, the relatively small sample size and the single-center nature of this study may have introduced biases. To address these limitations, future studies should aim to increase sample size, use higher-quality cases, and incorporate data from multiple centers. Such measures would enhance the rigor, accuracy, and generalizability of findings. We fully recognize these constraints and encourage further research to strengthen and validate our conclusions.

## Conclusion

In conclusion, gender, smoking after implant placement, oral hygiene status at the two-year follow-up of oral implant restoration, tooth position, number of implants, timing of loading, width of keratinized mucosa, and bone quantity were identified as factors influencing implant failure events in patients with dentition defects within two years of oral implant restoration. Among these, tooth position emerged as an independent factor, albeit with limited predictive power for implant failure in patients with partial dentition defects within this timeframe. The findings of this study may serve as a valuable reference for healthcare professionals in predicting implant failure events, enabling the development of targeted treatment and care plans with strong early warning potential.

## Data Availability

The simulation experimental data used to support the findings of this study are available from the corresponding author upon request.
